# Treatment of Hereditary Angioedema With Plasma‐Derived C1 Inhibitor: A Review

**DOI:** 10.1002/clt2.70126

**Published:** 2025-12-17

**Authors:** Inmaculada Martinez‐Saguer, Ingo Pragst, Beverley Worrall, Konrad Bork

**Affiliations:** ^1^ HZRM Hämophilie Zentrum Rhein Main GmbH Frankfurt Germany; ^2^ CSL Innovation GmbH Marburg Germany; ^3^ CSL Behring Innovation Pty Ltd Melbourne Australia; ^4^ Department of Dermatology University Medical Center Johannes Gutenberg University Mainz Germany

**Keywords:** C1 inhibitor, edema, hereditary angioedema, on‐demand treatment, prophylaxis

## Abstract

Hereditary angioedema (HAE) is clinically characterized by recurrent episodes of localized edema. HAE typically occurs due to a deficiency of functional C1 inhibitor (C1INH, HAE‐C1INH); in addition, several types of HAE with normal quantity and activity of C1INH (HAE‐nC1INH) have recently been classified, which occur due to different gene mutations. C1INH plays an integral role in the kallikrein–kinin system, where a deficiency of functional C1INH results in overproduction of bradykinin leading to subcutaneous and submucosal edema. Plasma‐derived C1INH (pdC1INH) replacement therapy for hereditary angioedema has been in use clinically for over 40 years and has been developed for both intravenous and subcutaneous administrations. In this review, we provide an in‐depth overview of the efficacy and safety of pdC1INH in clinical trials and real‐world studies, and guideline recommendations for pdC1INH replacement therapy as a first‐line treatment for on‐demand therapy, short‐term prophylaxis, and long‐term prophylaxis in patients with HAE‐C1INH Type 1 and 2, including special patient populations.

## 1 Background on Hereditary Angioedema

Hereditary angioedema (HAE) is a rare genetic disease that is characterized clinically by transient and recurrent episodes of localized edema. Clinical symptoms include swelling of the skin and tongue, attacks of abdominal pain, and laryngeal swelling which is potentially life‐threatening [1, 2]. There are multiple distinct types of HAE. HAE has classically been described as occurring due to a deficiency of functional C1 inhibitor (C1INH, HAE‐C1INH): HAE‐C1INH‐Type1 is caused by deficient plasma levels of C1INH (∼85% of HAE‐C1INH cases), and patients with HAE‐C1INH‐Type2 have normal plasma levels but dysfunctional C1INH (∼15% of HAE‐C1INH cases) [2]. More recently, various types of HAE with normal activity of C1INH (HAE‐nC1INH) have been classified, with six currently known underlying mutations [3, 4]: factor XII (HAE‐FXII) [1, 5], angiopoietin‐1 (HAE‐ANGPT1) [6], plasminogen (HAE‐PLG) [7], kininogen 1 (HAE‐KNG1) [8], myoferlin (HAE‐MYOF) [9], and heparan sulfate‐glucosamine 3‐O‐sulfotransferase 6 (HAE‐HS3ST6) [10]. However, there are further patients presenting with HAE‐nC1INH, in whom the genetic cause is unknown (HAE‐UNK) [3, 10]. The DAB2IP (disabled homolog 2 interacting protein) gene mutation has been described in a family with HAE and urticaria [11]. Similarly, the CPN (carboxypeptidase N) gene mutation has been identified in four families with HAE and urticaria [12].

The types of HAE‐nC1INH are considerably rarer than HAE‐C1INH, although the true prevalence of HAE‐nC1INH is difficult to estimate due to the challenges in diagnosing this type of HAE [13]. From here on, the term HAE is used in the text standing only for HAE due to C1INH deficiency (HAE‐C1INH or HAE Type 1 and 2), unless otherwise defined.

Patients with HAE Type 1 and 2 experience episodes of submucosal or subcutaneous (SC) edema (angioedema attacks) caused by the deficiency or dysfunction on C1INH leading to unregulated production of bradykinin and subsequent increased vascular permeability [14]. Attacks can last up to 5 days and can occur anywhere, but common sites include the patient's face, tongue, larynx, trunk, extremities, genitals, or gastrointestinal tract [2, 15]. During an attack, swelling can cause high levels of morbidity and mortality, especially in the case of laryngeal attacks that can result in asphyxiation [16]. Rates of mortality are higher in undiagnosed patients than in diagnosed ones, and in diagnosed patients, mortality is considered a rare event [17, 18]. Therefore, early recognition of HAE symptoms is vital to ensure effective and timely disease management [19].

However, long delays in HAE diagnosis are not uncommon, with the median time between patients experiencing their first attack and obtaining an accurate diagnosis ranging from 0.1 to 19 years and varying between countries [20, 21, 22, 23, 24, 25, 26]. Contributing factors to the diagnostic delay of HAE include the similarity of symptoms to more common conditions such as mast cell‐mediated angioedema and appendicitis, and low recognition and awareness of HAE among healthcare professionals (HCPs) due to the rarity of the disorder [27, 28]. Additionally, the clinical appearance and frequency of angioedema attacks are highly variable, both between patients and over the course of an individual patient's lifetime [29]. Consequently, patients are often misdiagnosed and may undergo unnecessary interventions, exacerbating diagnostic delay. In patients where HAE is suspected, it is recommended in the 2021 WAO/EAACI guideline that blood levels of C1INH function, C1INH protein and C4 should be assessed, if available, to accurately diagnose patients. The guidelines also recommend that family members (including children) should be screened for HAE, as HAE‐C1INH Types 1 and 2 should be suspected if there is a known family history with confirmed cases [2]. Screening where there is a positive family history can shorten diagnostic delay, even prior to symptom onset, and reduces the risk of mortality by asphyxiation during a laryngeal attack [17, 20].

## 2 The Multifaceted Role of C1INH

C1INH is a serine protease inhibitor, which plays a crucial role in multiple systems, most notably in the regulation of the complement system and the intrinsic coagulation (contact) system [15, 30]. In HAE‐C1INH, the contact system is initiated by auto‐activation of plasma protein factor XII (FXII) to FXIIa, with the subsequent cleaving of plasma prekallikrein (PK) to plasma kallikrein (PKa) [31]. The resultant PKa initiates the kallikrein–kinin system, where the cofactor high‐molecular‐weight kininogen (HK) is cleaved producing bradykinin [30, 31, 32]. FXIIa additionally activates the intrinsic coagulation system and fibrinolysis system [30, 32]. Bradykinin is the mediator of angioedema attacks in patients with HAE Type 1 and 2 [30]. The loss of functional C1INH leads to the overproduction of bradykinin and its binding to bradykinin B2 receptor on endothelial cells, initiating vasodilation and increasing vascular permeability and plasma leakage, causing subcutaneous and submucosal edema.

**FIGURE 1:**
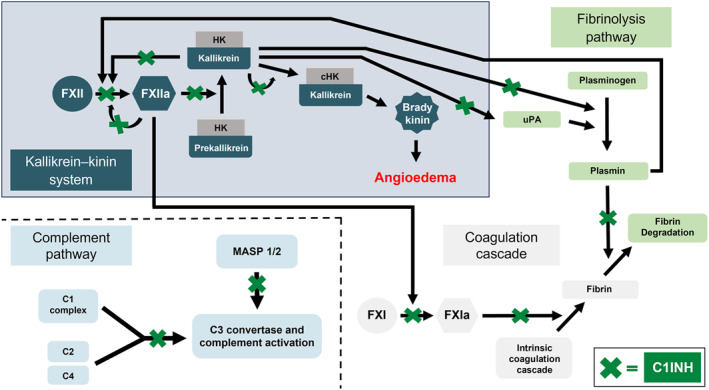
The pathway of HAE‐C1INH and the additional targets of C1INH [35, 36]. (Adapted from Petersen et al. 2024, and Cohn and Renné 2024, under the Creative Commons Attribution License (https://creativecommons.org/licenses/by/4.0/)). The kallikrein–kinin system is initiated when plasma protein factor XII (FXII) is activated to FXIIa on contact with negatively charged surfaces. FXIIa cleaves plasma prekallikrein to kallikrein, and kallikrein cleaves the cofactor high‐molecular‐weight kininogen, producing bradykinin. Positive feedback loops result in increased FXIIa. In HAE‐C1INH Types 1 and 2, deficiency or dysfunction of C1INH lead to over production of bradykinin and result in angioedema. The intrinsic coagulation cascade and fibrinolysis pathway are also activated by FXII activation, and C1INH is involved in the regulation of both systems, as well as the complement pathway, where C1INH inhibits the classical pathway (C1 complex, C2, C4) and the lectin pathway (MASP 1/2). C1, complement 1; C1INH, C1 inhibitor; C2, complement 2; C4, complement 4; cHK, cleaved HK; HK, high‐molecular‐weight kininogen; MASP, mannose‐binding lectin‐associated serine protease; uPA, urokinase plasminogen activator.

In addition to the contact system, C1INH regulates both the classical and lectin pathways of the complement system [30]. A loss of functional C1INH is associated with increased activation of C1 and depletion of C2 and C4 complement components [33]. The complement system plays a key role in the regulation of innate and adaptive immunity, including defense against microbial infections [34]. The multiple inhibitory targets of C1INH in these pathways, which are known to interact, demonstrate its complex regulatory role (Figure 1) [35, 36].

The influence of C1INH deficiency on bradykinin‐mediated HAE attacks is well described; however, the effect of complement dysregulation due to C1INH deficiency in patients with HAE is less well known. Studies have shown that dysfunction of the complement system is associated with increased risk of infection, hypertension and autoimmune disease [34, 37, 38, 39, 40]. In line with this, a higher prevalence of comorbidities such as autoimmune diseases have been observed in patients with HAE Type 1 and 2 compared with the general population, of which systemic lupus erythematosus (SLE) is the most commonly reported [37, 39]. The depletion of early complement components seen with C1INH deficiency has been associated with increased risk of SLE and glomerulonephritis [37, 41, 42]. In addition to autoimmune disease, some studies have demonstrated that patients with HAE are also at a greater risk of cardiovascular disease, endothelial dysfunction, hypertension and hyperlipidemia compared with individuals without HAE [39, 43, 44], although the evidence is not conclusive [45]. High levels of plasma C1INH are also associated with a lower risk of venous thromboembolism (VTE), and patients with HAE caused by deficient or dysfunctional C1INH have been demonstrated to have an increased risk of composite VTE [43, 46]. Currently, there is some initial evidence that C1INH replacement therapy may reduce the risk of comorbid conditions in patients with HAE Type 1 and 2 and reduce hospital visits [47, 48]; however, further evidence is needed.

## 3 The History of Plasma‐Derived C1INH in the Treatment of HAE

Before the introduction of HAE‐specific therapy, laryngeal HAE attacks were often fatal, and in undiagnosed patients, asphyxiation is still a significant cause of mortality [17, 18, 49]. However, there are now several drugs available that target the pathways to bradykinin‐mediated angioedema that are effective at relieving symptoms of an attack (on‐demand treatment) or preventing/reducing the number of attacks (prophylaxis). The first use of purified plasma‐derived C1INH (pdC1INH) replacement therapy for the treatment of HAE attacks was published in 1973 [50]. This led to further studies demonstrating the efficacy of pdC1INH therapy in reducing attack severity, and subsequently Berinert (CSL Behring), an intravenous (IV) pdC1INH therapy, was the first treatment approved for acute on‐demand treatment of HAE attacks in Europe in 1979 [51, 52]. In 2008, Berinert IV completed the European Mutual Recognition Procedure for on‐demand treatment of acute attacks in all age groups, and in 2009 received FDA approval for on‐demand use in the United States. Cetor (pdC1INH; Sanquin) was also made available for patients with HAE and angioedema due to acquired C1INH deficiency in 1997; however, manufacture was ceased in 2012 in favor of Cinryze (IV pdC1INH; Takeda) [53, 54]. Cinryze was approved for routine prophylaxis in the United States in 2008 and for the acute treatment of HAE attacks, routine prophylaxis and pre‐procedural (short‐term) prophylaxis in Europe in 2011, followed by an extended label to include children ≥ 6 years old for routine prophylaxis in Europe in 2017 [55].

A label expansion for self‐administration of Berinert IV was approved in 2011 by the EMA and 2012 by the FDA. Reported benefits of self‐administration include improved health‐related quality of life (HRQoL), reduced need for admission to hospitals for acute attacks and related costs, and increased patient confidence [56]. Shortly after the label expansion for self‐administration, Berinert IV was granted EMA approval in 2013 for pre‐procedural (short‐term) prophylaxis prior to medical, dental or surgical procedures to prevent HAE attacks.

Following the success of IV pdC1INH treatment, alternative administration methods of pdC1INH were considered. A high concentration, reduced volume formulation was investigated for subcutaneous (SC) use in 2015 [57], and in 2017, the first SC pdC1INH for routine prophylaxis was approved for use in patients 6 years of age and older with HAE (HAEGARDA [USA], CSL Behring) and for use in adults and adolescents with HAE (Berinert 2000/3000 [Europe], CSL Behring); which allows for a more convenient self‐administration by the patient or caregiver [58]. The introduction of SC pdC1INH removed the need for venous access and indwelling ports, and therefore reduced the associated risk of infections, whilst the reduced volume also shortened the time taken to administer [55]. Subcutaneous administration also maintains more stable steady‐state levels of C1INH, increasing minimum trough levels in pharmacokinetic simulations, and improving efficacy by providing greater prevention from HAE attacks when compared with IV administration in indirect comparisons and switch studies [55, 57, 59].

Human pdC1INH is a direct replacement of the reduced functional endogenous C1INH of patients with HAE Type 1 and 2, restoring regulation of bradykinin release and attenuating or preventing angioedema attacks [2, 60, 61, 62]. Although viral transmission is a potential risk when using plasma‐derived products [63, 64], the manufacturing processes of pdC1INH includes multiple steps that reduce the risk of virus transmission and enhance the purity of the end concentrate [64], and as such there have been no confirmed reports of viral transmissions from the use of Berinert or HAEGARDA [60, 65, 66].

## 4 Pivotal Trial Data With Plasma‐Derived C1INH

### 4.1 On‐Demand

The efficacy and safety of IV pdC1INH for the treatment of acute HAE attacks was evaluated between 2005 and 2007 in the Phase 2/Phase 3 I.M.P.A.C.T.1 trial (a randomized, three‐arm, double‐blind, placebo‐controlled study using C1INH Berinert) [60]. A dose of 20 IU/kg pdC1INH, compared with placebo, significantly reduced the median time to symptom relief after attack, from 1.5 to 0.5 h (*p* = 0.0025) and significantly reduced the median time to complete symptom relief (*p* = 0.0237) [60]. Patients also experienced fewer adverse events within 4 hours after pdC1INH treatment compared with placebo (19.6% in 20 IU/kg pdC1INH vs. 43.9% in placebo), and no patient experienced serious adverse events in the first 4 hours of treatment [60].

Based on the positive data from the I.M.P.A.C.T.1 trial, a prospective, open‐label, uncontrolled, multicenter extension Phase 3 study, I.M.P.A.C.T.2, was conducted between 2005 and 2010, that reported long‐term efficacy and safety of on‐demand pdC1INH Berinert (20 IU/kg) across 1085 HAE attacks [65]. The I.M.P.A.C.T.2 open‐label extension (OLE) also demonstrated that 20 IU/kg pdC1INH had a fast onset of action, with a per‐patient median time to symptom relief of 0.46 and 15.5 h to complete symptom resolution; for laryngeal attacks, per‐patient median time to symptom relief was 0.44 and 5.79 h to complete symptom resolution [65]. In addition, only 1/1085 attacks had a rebound attack, and a single dose was sufficient to effectively treat 99% of attacks [65]. The most common adverse events reported across both the I.M.P.A.C.T trials were headaches, nausea, diarrhea, pain (most frequently abdominal pain), muscle spasms and nasopharyngitis [60, 65].

A further randomized double‐blind, placebo‐controlled Phase 3 trial using the on‐demand IV pdC1INH therapy Cinryze also demonstrated that patients experienced a significantly reduced time to onset of symptom relief (4 h with placebo to 2 h with 1000 IU pdC1INH [*p* = 0.02]), with significantly reduced time to complete resolution of symptoms (*p* = 0.004) [61]. In this study, only 1/36 patients receiving on‐demand IV pdC1INH Cinryze (1000 IU) experienced a treatment‐related adverse event, which was a rash at the injection site, of mild severity [61].

In the aforementioned studies, re‐dosing was required in 6/57 patients (1% of total overall attacks) with pdC1INH Berinert IV and in 23/35 patients with pdC1INH Cinryze; the difference may be due to a weight‐based dosing regimen compared with a fixed‐dose regimen, respectively [61, 65]. This is demonstrated in an indirect comparison study of clinical outcomes, based on a systemic literature search, which reviewed weight‐based and fixed dosing [67]. Patients treated with body‐weight‐adjusted doses of on‐demand pdC1INH experienced significantly reduced median times for symptom relief (26.1 vs. 42.5 min, *p* = 0.019) and complete resolution of laryngeal attacks (10.5 vs. 17.8 h, *p* = 0.037) where redosing was not necessary compared with patients in the fixed‐dose cohort [67].

Studies have shown that self‐administration of on‐demand IV pdC1INH treatment is associated with improved HRQoL measures in patients with HAE. This was observed in a small study of seven patients who experienced frequent or severe debilitating HAE attacks, which had negative impacts on their HRQoL [68]. After the introduction of on‐demand self‐administration of IV pdC1INH, their Dermatology Life Quality Index (DLQI) was significantly improved (*p* < 0.001) and HRQoL as measured by SF‐36 was also significantly improved in all domains [68]. A Danish study in 2014 found similar results after the introduction of home‐based therapy, which included on‐demand pdC1INH, where the number of patients who experienced significant psychological impact due to HAE reduced after the introduction of home‐based therapy [69].

The option for at‐home administration of on‐demand IV pdC1INH for acute attacks can prevent treatment delays and therefore allow for better treatment outcomes, such as a decreased time to initial symptom relief, and decreases in the number of days in hospital [70]. Self‐administration provides patients with greater freedom and flexibility to administer at home and avoids unnecessary costs associated with healthcare visits [56]. In addition, patients can maintain personal safety and minimal disruption in living a healthy and productive life including fewer days missed from school and work [68, 70]. Accordingly, the 2021 WAO/EAACI guideline recommends that “all patients who are provided with on‐demand treatment licensed for self‐administration should be taught to self‐administer” [2].

### 4.2 Short‐Term Prophylaxis (STP)

In addition to patient‐specific triggers, medical procedures including dental procedures and surgery can trigger attacks in patients with HAE, with a risk of perioperative attack of up to 30.5% [71, 72]. Thus, STP is recommended before these procedures and events [2]. STP with IV pdC1INH has been demonstrated to reduce the incidence of angioedema attacks in both adults and children [71, 73, 74]. Patients treated with C1INH STP before surgical interventions have reduced post‐procedure HAE attacks [73, 75], to a greater extent than tranexamic acid and danazol [73], and is associated with a reduced risk of HAE symptoms after dental procedures [71, 76].

The Berinert patient registry, conducted between 2010 and 2014 at 30 US and European sites, represents the largest real‐world evaluation of pdC1INH use and one of few data sets that report its use for STP. Data from the registry demonstrated that around 25% of patients had used IV C1INH for STP at least once and the cumulative rate of HAE attacks was low at 0.04, 0.06 and 0.11 attacks per infusion within 1, 2 and 3 days of infusion, respectively [74].

### 4.3 Long‐Term Prophylaxis (LTP)

Plasma‐derived C1INH is recommended as a first‐line LTP treatment for the prevention of HAE attacks [2]. In a randomized double‐blind, placebo‐controlled Phase 3 trial, LTP with Cinryze (1000 IU) reduced the frequency of HAE attacks by ∼50% when compared to placebo (*p* < 0.001), with no treatment‐related serious adverse events reported in the Phase 3 trial [61]. The safety of the pdC1INH Cinryze (1000 IU) has also been demonstrated in an open‐label multicenter extension study where 146 patients received treatment for up to 2.6 years. The study found no severe hypersensitivity reactions related to treatment and found no evidence of anti‐C1INH antibodies [77].

Prophylactic treatment with SC pdC1INH (HAEGARDA/Berinert 2000/3000) results in sustained C1INH protein levels and is well tolerated [78, 79]. This was shown in the COMPACT Phase 3 trial between 2013 and 2015. The study utilized a crossover design where patients were randomized into four groups and received a 16‐week treatment of SC pdC1INH (40 IU or 60 IU of SC C1INH per kg of body weight) twice weekly followed by a 16‐week treatment with placebo, or vice versa [62]. The study demonstrated that twice‐weekly prophylactic SC pdC1INH with a 60 IU/kg dose led to a 95% median reduction in attack rate and a 100% median reduction in rescue medication use [62].

Subsequently, the COMPACT Phase 3 OLE trial showed both 40 IU/kg and 60 IU/kg doses of SC pdC1INH (HAEGARDA/Berinert 2000/3000) were well tolerated, with no dose‐dependent safety concerns after long‐term use (mean duration of use of 1.5 years) [66]. The most common adverse events reported in the COMPACT trials were injection‐site reactions of mild severity and no serious adverse events assessed as causally related to the treatment or neutralizing antibodies were observed [66]. Of the patients who were prescribed 60 IU/kg, 62% required no rescue medication per year resulting in a median use of rescue medication of 0.0 uses per year [66]. Additionally, over the course of the study the percentage of patients that were attack free increased, suggesting that there was no tolerance induction. More recently a US extension of the study found that in 23 patients receiving SC C1INH prophylaxis for > 2 years, 87% did not require rescue medication, and 83% were attack free during the week 25–30 observation period [80]. Results from additional analyses, based on patients switching from IV pdC1INH to SC pdC1INH prophylaxis in the COMPACT Phase 3 trial, or from indirect treatment comparisons, have complemented these findings and have demonstrated that SC pdC1INH had greater efficacy than IV treatment, with patients experiencing greater attack reduction and prevention [55, 59].

Although prophylactic HAE treatments show high efficacy, the occurrence of occasional breakthrough attacks requiring treatment is a possibility [77, 81]. Severe attacks require urgent intervention and may require emergency department visits or hospitalization. In the COMPACT Phase 3 study, no patients receiving 60 IU/kg SC pdC1INH experienced a laryngeal attack, and attack severity was also reduced compared to placebo [62]. Additionally, population pharmacokinetic analysis of SC and IV pdC1INH found that C1INH functional activity levels remained consistently above the clinically meaningful threshold of 40% with SC pdC1INH administration, with consistently higher trough levels compared with IV route of administration [79].

In addition to improved convenience and efficacy of SC pdC1INH compared with IV pdC1INH [55], prophylactic SC pdC1INH has also shown positive impacts on HRQoL. Post‐hoc analyses of the COMPACT Phase 3 trial evaluated HRQoL after twice‐weekly prophylactic treatment with SC pdC1INH and found improvements in Work Productivity and Activity Impairment (WPAI) scores compared with placebo [82]. Furthermore, improvements in HRQoL were also observed in the COMPACT Phase 3 OLE study [83], which found that, despite 63% of patients already having received LTP with pdC1INH prior to recording baseline HRQoL measures, there remained a significant improvement in multiple HRQoL measures in those receiving 60 IU/kg pdC1INH LTP. This included significant improvements in the Hospital Anxiety and Depression Scale (HADS), WPAI and angioedema‐specific tools—AE‐QoL and HAE‐QoL [83]. A qualitative research study on 14 patients receiving SC pdC1INH for ≥ 3 months detailed the effect of the treatment on their life [84]. These included: not feeling limited by HAE, improved ability to travel, ease of treatment administration, increased confidence, improved sleep and energy, healthier family relationships and improved cognition [84]. Additionally, a recent study of 737 patients found that the use of any form of prophylaxis resulted in a clinically meaningful improvement of 33.3% in median AE‐QoL scores, relative to on‐demand‐only treatment [85].

## 5 Safety Overview

The first approval for use of Berinert as an IV injection was in Germany, 1979, and consequently human C1INH concentrate has been used clinically for over 40 years [51]. Between 1985, when Berinert was launched, and 2011, when the I.M.P.A.C.T. 2 Phase 3 OLE was published, it is estimated that 630,000 treatments of Berinert were administered globally [30], and since 2011 new products have been developed and indications have expanded within HAE, to include STP and LTP. During the 40 years of clinical use of pdC1INH, there have been multiple clinical trials, including on‐demand and LTP usage, and with IV and SC administrations, in which pdC1INH has been demonstrated to be well tolerated [60, 61, 62, 65, 66, 77, 78]. Consequently, the safety profile of pdC1INH means it is recommended for multiple special populations (discussed in detail in the section dedicated to special populations), and is the only treatment recommended in the World Allergy Organization (WAO) and the European Academy of Allergy and Clinical Immunology (EAACI) guideline for HAE‐C1INH during pregnancy and lactation [2].

Since C1INH naturally occurs in the blood of healthy patients, pdC1INH has a favorable safety profile in comparison to alternative treatments such as the androgens, which have been associated with more serious adverse events across HAE populations [2]. Plasma‐derived products do have a potential risk of viral transmission, but to date, there are no confirmed reports in the scientific literature of viral transmissions from the use of Berinert or HAEGARDA [32, 60, 65, 66]. Hypersensitivity reactions with use of pdC1INH have been observed, and given that hypersensitivity reactions may present with symptoms similar to an attack, patients should be informed as to how to recognize the early signs of hypersensitivity [62, 86].

Instances of thrombosis have been reported in the scientific literature with use of pdC1INH, but when used as per their respective licensed indications and recommended doses, a causal relationship between thromboembolic events and the use of Berinert IV and Berinert SC has not been established [32, 87, 88, 89]. To understand the relative risk factors relating to thromboembolism, a long‐term follow‐up study monitored patients using Berinert IV and concluded that in therapeutic doses for HAE‐C1INH, Berinert IV is not associated with increased rates of thromboembolism when compared with patients not treated with Berinert IV [88]. In this study, it was observed that a large proportion of affected patients used IV indwelling ports, which may be a risk factor for thrombosis [55, 87, 88]. More recently, a post‐hoc analysis of the Phase 3 COMPACT trial indicated that median D‐dimer coagulation parameters were attenuated with SC pdC1INH therapy, indicating better vascular system stability with pdC1INH prophylaxis [90]. This would be consistent with observations that patients with HAE caused by deficient or dysfunctional C1INH have an increased risk of composite VTE, and that high levels of pdC1INH are associated with lower risk of VTE [43, 46].

## 6 Current HAE‐C1INH Type 1 and 2 Treatment Guideline Recommendations

The combined efficacy, safety and HRQoL data has resulted in pdC1INH being recommended as a first‐line LTP, STP and on‐demand therapy for HAE‐C1INH Types 1 and 2, in international HAE treatment guidelines [2, 91]. The current recommendations for first‐line treatments, according to the WAO/EAACI guideline, are summarized in Table 1 [2, 58, 89, 92, 93, 94, 95, 96, 97, 98, 99, 100, 101, 102, 103, 104]. It should be noted that not all treatments recommended in these guidelines are approved in all countries or jurisdictions; there are regional variabilities in approvals. In addition, new treatments targeting specific single proteins of the contact system (e.g., FXIIa, prekallikrein or plasma kallikrein), have also recently been launched in 2025, which are not included in the WAO/EAACI guidelines (updated in 2021), these include garadacimab and donidalorsen for LTP and sebetralstat for on‐demand treatment [105, 106, 107, 108, 109, 110].

**TABLE 1:** WAO/EAACI guideline recommendations regarding recommended first‐line treatments in HAE Type 1 and 2.

WAO/EAACI guideline treatment recommendations [2]		
Treatment: Brand	Administration	Age
On‐demand (ODT) recommended treatments [89, 92, 93, 94, 95, 96, 97, 98] (It is recommended that all attacks are considered for on‐demand treatment)		
pdC1INH: Berinert 500/1500^a^	IV (self‐administer)	Any
pdC1INH: Cinryze^b^	IV (self‐administer)	≥ 2 years old
rhC1INH: Ruconest^c^	IV (self‐administer, US)	≥ 13 years old (US)
	IV (HCP, Europe)	≥ 2 years old (Europe)
Ecallantide (only US): Kalbitor^d^	IV (HCP)	≥ 12 years old
Icatibant: Firazyr (and generics)	SC (self‐administer)	≥ 2 years old
Short‐term prophylaxis (STP) recommended treatments [89, 92, 96] (STP is recommended before medical, surgical, or dental procedures, as well as exposure to other attack‐inducing events)		
pdC1INH: Berinert 500/1500^a^ ^,^ ^e^	IV (self‐administer)	Any
pdC1INH: Cinryze^b^	IV (self‐administer)	≥ 2 years old
Long‐term prophylaxis (LTP) recommended treatments [58, 96, 99, 100, 101, 102, 103, 104] (It is recommended that the goals of treatment are to achieve total control of the disease and to normalize patients' lives)		
pdC1INH: Berinert 2000/3000^f^	SC (self‐administer)	≥ 12 years old
pdC1INH: HAEGARDA^f^	SC (self‐administer)	≥ 6 years old
pdC1INH: Cinryze	IV (self‐administer)	≥ 6 years old
Lanadelumab: Takhzyro	SC (self‐administer)	≥ 2 years of age
Berotralstat: Orladeyo	Oral (self‐administer)	≥ 12 years of age

*Note:* All pdC1INH concentrates are licensed for self‐administration; however, approved product indications may vary at the country/regional level. Guidelines recommend that all patients who are provided with on‐demand treatment licensed for self‐administration should be taught to self‐administer [2].

Abbreviations: FDA, US Food and Drug Administration; HCP, healthcare professional; IV, intravenous; LTP, long‐term prophylaxis; pdC1INH, plasma‐derived C1‐esterase inhibitor; rhC1INH, recombinant human C1‐esterase inhibitor; SC, subcutaneous; STP, short‐term prophylaxis; US, United States.

^a^ Only Berinert 500 is approved for use in the United States by the FDA.

^b^ Cinryze is not indicated in the United States by the FDA for on‐demand treatment or STP.

^c^ Ruconest is indicated in the United States for self‐administration and for aged ≥ 13 years old, whereas in Europe it is indicated for aged ≥ 2 years old and to be administered by a healthcare professional.

^d^ Kalbitor is indicated in the United States, but not Europe, for on‐demand treatment.

^e^ Berinert 500 is not indicated for STP in the United States.

^f^ HAEGARDA is the brand name used in the United States and selected other countries, Berinert 2000/3000 the brand name used in Europe.

The availability of new LTP therapy options for HAE led to a Delphi consensus that redefined the ultimate goals of treatment for HAE. The authors agreed that the ultimate goals of treatment in HAE are to achieve total control of the disease and to normalize the patient's life [111]. The WAO/EAACI guideline for the management of HAE reflect these goals, and also recommends these two treatment goals (100% agreement; evidence level D) [2]. The Delphi panel agreed on factors to consider when determining whether a patient's disease is controlled, and/or their life is normalized. These factors included attack frequency, the requirement for rescue medication, length of attack‐free periods, Emergency Department visits, number of days of sick leave, and the number of hours of activity impairment [111].

To date, the treatment recommendations for HAE‐nC1INH are based on individual reports and case series and include the use of pdC1INH, progesterone, tranexamic acid and danazol; there is some evidence to suggest pdC1INH may be effective in HAE‐nC1INH both with FXII mutation and plasminogen mutation [112, 113, 114]. Further research into these subtypes of HAE will help determine the most appropriate treatment options for these patients. A recent consensus paper for the treatment of HAE‐nC1INH, based on expert opinion, outlines best‐practice approaches for the different types of HAE‐nC1INH [4].

## 7 Plasma‐Derived C1INH for the Treatment of Special Populations

The safety and efficacy of pdC1INH for the treatment of acute attacks in special populations has also been evaluated, including during pregnancy, lactation, and childhood [115, 116, 117]. Specific considerations to treat these special populations have been acknowledged in several studies and in evidence‐based recommendations for the therapeutic management of HAE [118, 119]. Treatment plans during pregnancy and delivery should be individualized to ensure the safety for both mother and the fetus/newborn, and the patient's care should be shared with a clinician experienced in the management of HAE [120]. Evidence suggests that pdC1INH treatment during pregnancy does not affect the rate of spontaneous abortion, the prevalence of obstetrical syndromes, or neonatal short‐term outcomes [115]. A post‐hoc subgroup analysis of the COMPACT Phase 3 trial was performed on women aged between 15 and 45 years, including four women who became pregnant during the study [121]. This analysis found that SC pdC1INH (HAEGARDA/Berinert 2000/3000) was effective and supported for safe use in women of childbearing age with a 95% median reduction in attack frequency compared with before the study, and all four pregnancies that occurred during the study had no complications. [121] In addition, a retrospective case collection study including 22 pregnant women with HAE‐C1INH Type 1 (aged 20–38 years; 35 pregnancies) received IV pdC1‐INH doses of 500 or 1000 IU for the treatment of acute attacks before, during, and/or after pregnancy, and no adverse events were associated with pdC1INH treatment [116]. Similarly, no adverse events were associated with pdC1INH treatment in observational registry data collected during 11 pregnancies (10 females; aged 16–40 years) receiving up to 3000 IU IV pdC1INH [119]. As such, the current WAO/EAACI guideline recommends pdC1INH as the preferred treatment during pregnancy and breastfeeding for acute attacks, preprocedural STP, and LTP [2]. However, the data available is limited, and although this limited data suggests no increased risk from the use of pdC1INH, treatment with IV pdC1INH in pregnancy is only advised if there is a clinical need [89, 92, 96, 104]. Similarly, limited data suggest no increased risk from the use of SC pdC1INH in pregnant women. Given that C1INH is a physiological component of human plasma, no adverse effects on pre‐ and postnatal development are expected [58, 101]. Further, there is no information regarding the excretion of C1INH in human milk, therefore the healthcare professional is advised to consider the individual patient and her child's development and health benefits along with the clinical need for C1INH [58, 89, 92, 96, 101, 104].

In pediatric populations, pdC1INH treatment can also be used for HAE treatment; similar to results in adult populations, a study including 37 pediatric patients who received pdC1INH as on‐demand or prophylactic treatment found no adverse events and no cases of anti‐C1INH‐antibodies [122]. The approved C1INH treatments for children and adolescents are summarized in Table 1 [2, 58, 89, 92, 93, 94, 95, 96, 97, 98, 99, 100, 101, 102, 103, 104], note that age ranges for approval vary between different indications, methods of administration and regulatory authority [58, 89, 92, 96, 101, 104]. On‐demand and STP use of IV pdC1INH is currently the only treatment approved for children under the age of 2 years [89].

Plasma‐derived C1INH is also supported for safe use in older patients. In a study of patients ≥ 65 years old, the rates of adverse events in patients ≥ 65 years were similar to younger adults (0.02 and 0.03 per infusion, respectively) [123]. Additionally, 94.6% of infusions were administered outside of a healthcare setting and there was no evidence of difficulties with self‐administration in the older population [123]. A post‐hoc subgroup analysis of COMPACT OLE showed that long‐term treatment with SC pdC1INH in the COMPACT trial was effective and well tolerated in patients ≥ 65 years old (*n* = 10), with 60% of patients experiencing < 1 attack per month, despite a number of these patients having comorbidities and taking more than five concomitant non‐HAE‐related drugs [124].

## 8 Summary of pdC1INH Product Characteristics

The currently available pdC1INH treatments are: Berinert, IV administration; Cinryze, IV administration and Berinert/HAEGARDA, SC administration [58, 89, 92, 96, 101, 104]. Their recommended indications are tabulated in Table 1 [2, 58, 89, 92, 93, 94, 95, 96, 97, 98, 99, 100, 101, 102, 103, 104]; however, further details relating to dosing and storage are included in Table 2 [58, 89, 92, 96, 101, 104]. This summary highlights the different indications (on‐demand, STP and LTP) for pdC1INH across the HAE‐C1INH population. Both Berinert and Cinryze are well‐established therapeutics for HAE, but new pdC1INH therapies are also in clinical development with OCTA‐C1‐INH (Octapharma) in Phase 3 trials [58, 89, 92, 96, 101, 104, 125, 126].

**TABLE 2:** Overview of product characteristics of approved pdC1INH products.

pdC1INH product	Approval for use
Berinert 500/1500^a^ [89, 92]	On‐demand: 20 IU/kg
Administration: IV	STP: 1000 IU, < 6 h prior to medical, dental, or surgical procedure; 15–30 IU/kg (pediatric population)^b^
Store: < 30° (do not freeze)	
Cinryze [96, 104]	On‐demand: 1000 IU; 500 IU (2–11 years and 10–25 kg)^b^ ^,^ ^c^
Administration: IV	STP: 1000 IU, < 24 h prior to medical, dental, or surgical procedure; 500 IU (2–11 years and 10–25 kg)^b^ ^,^ ^c^
Store: < 25° (do not freeze)	LTP: 1000 IU, every 3–4 days; 500 IU, every 3–4 days (6–11 years)
Berinert 2000/3000 (Europe) [101]; HAEGARDA (US) [58]	LTP: 60 IU/kg, every 3–4 days
Administration: SC	
Store: < 30° (do not freeze)	

Abbreviations: FDA, US Food and Drug Administration; IU, international unit; IV, intravenous; kg, kilogram; LTP, long‐term prophylaxis; ODT, on‐demand treatment; SC, subcutaneous; STP, short‐term prophylaxis.

^a^ Only Berinert 500 is approved for use in the United States by the FDA.

^b^ Not currently approved for this indication in the United States by the FDA.

^c^ Although the international WAO/EAACI guidelines for the management of hereditary angioedema (2021 revision and update) note that most experts use either 1000 IU or 20 IU/kg for STP, the manufacturer only recommends weight‐based dosing with Berinert 500/1500 for the pediatric population, with the fixed 1000 IU dose intended for STP in the adult population only [2, 89]. For Cinryze, the recommended pediatric dose is reduced to 500 IU [96].

## 9 The Value of pdC1INH in the Emerging HAE Treatment Landscape

Plasma‐derived C1INH remains a cornerstone in the management of HAE‐C1INH due to its unique mechanism of replacing the deficient or dysfunctional endogenous inhibitor, broad indication coverage (on‐demand, STP and LTP) and favorable safety profile in patients of all ages, including vulnerable populations [60, 61, 62, 65, 66, 77, 78, 118, 119, 122, 123]. Since the first approval of IV pdC1INH therapy for acute on‐demand treatment in Europe in 1979, there have been multiple label expansions [51, 52]. Cinryze was approved for routine prophylaxis in the United States in 2008, preparing the field for prophylactic treatment options for HAE‐C1INH [55, 61, 104]. Subsequently, greater reductions in attack rate were observed in studies with SC pdC1INH (HAEGARDA/Berinert 2000/3000) which helped to raise the expectation of what prophylactic treatments in HAE‐C1INH could achieve, contributing to the formation of the goal that LTP should aim to achieve total control of the disease and to normalize the patient's life [58, 62, 111].

The landscape of HAE therapeutic interventions has evolved quickly since 2017 and continues to develop. Due to the nature of pdC1INH, it still has an important role as a treatment option. All other on‐demand and LTP treatments for HAE‐C1INH in Table 1, as well as other newly approved treatments, target proteins in the contact system or kallikrein–kinin system that lead to the overproduction of bradykinin when there is a lack of functional C1INH to provide regulation. The value of pdC1INH in the evolving therapeutic landscape is its role in regulation across multiple systems (e.g., the contact, complement and coagulations systems), as well as the kallikrein–kinin system (Figure 1), which may have advantages in complex clinical scenarios.

Extensive use in clinical practice, along with clinical trial data, have demonstrated pdC1INH to be well tolerated [60, 61, 62, 65, 66, 77, 78]. It is unique as a therapeutic intervention for HAE‐C1INH in being indicated for on‐demand, STP and LTP. Consequently, it has particular value in special populations, as previously discussed, with the WAO/EAACI guideline recommending pdC1INH as the preferred treatment during pregnancy and breastfeeding [2]. It also has specific value as a STP to derisk the occurrence of attacks during medical procedures, where surgery and dental procedures can induce angioedema attacks [2].

## 10 Conclusion

As part of an expanded patient treatment choice, pdC1INH still retains a critical role for patients requiring broad systemic regulation, favorable safety demonstrated in vulnerable populations, or those preferring a replacement therapy approach. The replacement of deficient C1INH through administration of pdC1INH concentrate represents a direct treatment approach to regulate bradykinin production and prevent HAE attacks. Several studies and patient cases, along with over 40 years of use in routine clinical practice, have demonstrated that pdC1INH therapy is well tolerated and effective in patients of all ages and has a favorable safety profile. In the recent 2022 WAO/EAACI guideline, pdC1INH was recommended as a first‐line treatment for on‐demand therapy, STP, and LTP in patients with HAE Type 1 and 2. Furthermore, pdC1INH is recommended as the preferred treatment for the management of HAE Type 1 and 2 during pregnancy and lactation. The fast onset of relief and low requirement for redosing with on‐demand use, and the low risk of breakthrough attacks during prophylactic use, along with the established safety profile and HRQoL improvements, demonstrate the consistent clinical benefit of therapy with pdC1INH for the treatment of HAE‐C1INH.

## Author Contributions

**Inmaculada Martinez‐Saguer:** conceptualization, writing – review and editing. **Ingo Pragst:** writing – review and editing. **Beverley Worrall:** writing – review and editing. **Konrad Bork:** conceptualization, writing – review and editing.

## Conflicts of Interest

Medical writing support and publication was funded by CSL Behring. I.M.S. received speaker/consultancy fees and funding to attend conferences/educational events from CSL Behring, Takeda, BioCryst, Octapharma, Pharvaris and KalVista. She participated as investigators (PI/SI) in a clinical trial/registry for CSL Behring, BioCryst, Octapharma, KalVista, Pharvaris and Takeda. I.P. is an employee of CSL Innovation GmbH and shareholder of CSL limited. B.W. is an employee and shareholder of CSL limited. K.B. has received research grants and/or lecture fees from CSL Behring and Takeda for unrelated projects.

## Data Availability

Data sharing not applicable to this article as no datasets were generated or analyzed during the current study.

## References

[1] G. Dewald and K. Bork , “Missense Mutations in the Coagulation Factor XII (Hageman Factor) Gene in Hereditary Angioedema With Normal C1 Inhibitor,” Biochemical and Biophysical Research Communications 343, no. 4 (2006): 1286–1289, 10.1016/j.bbrc.2006.03.092. 16638441

[2] M. Maurer , M. Magerl , S. Betschel , et al., “The International WAO/EAACI Guideline for the Management of Hereditary Angioedema—The 2021 Revision and Update,” Allergy 77, no. 7 (2022): 1961–1990, 10.1111/all.15214. 35006617

[3] A. Reshef , T. Buttgereit , S. D. Betschel , et al., “Definition, Acronyms, Nomenclature, and Classification of Angioedema (DANCE): AAAAI, ACAAI, ACARE, and APAAACI DANCE Consensus,” Journal of Allergy and Clinical Immunology 154, no. 2 (2024): 398–411.e391, 10.1016/j.jaci.2024.03.024. 38670233

[4] B. L. Zuraw , K. Bork , L. Bouillet , et al., “Hereditary Angioedema With Normal C1 Inhibitor: An Updated International Consensus Paper on Diagnosis, Pathophysiology, and Treatment,” Clinical Reviews in Allergy and Immunology 68, no. 1 (2025): 24, 10.1007/s12016-025-09027-4. 40053270 PMC11889046

[5] K. Bork , K. Wulff , P. Meinke , N. Wagner , J. Hardt , and G. Witzke , “A Novel Mutation in the Coagulation Factor 12 Gene in Subjects With Hereditary Angioedema and Normal C1‐inhibitor,” Clinical Immunology 141, no. 1 (2011): 31–35, 10.1016/j.clim.2011.07.002. 21849258

[6] V. Bafunno , D. Firinu , M. D'Apolito , et al., “Mutation of the Angiopoietin‐1 Gene (ANGPT1) Associates With a New Type of Hereditary Angioedema,” Journal of Allergy and Clinical Immunology 141, no. 3 (2018): 1009–1017, 10.1016/j.jaci.2017.05.020. 28601681

[7] K. Bork , K. Wulff , L. Steinmuller‐Magin , et al., “Hereditary Angioedema With a Mutation in the Plasminogen Gene,” Allergy 73, no. 2 (2018): 442–450, 10.1111/all.13270. 28795768

[8] K. Bork , K. Wulff , H. Rossmann , et al., “Hereditary Angioedema Cosegregating With a Novel Kininogen 1 Gene Mutation Changing the N‐Terminal Cleavage Site of Bradykinin,” Allergy 74, no. 12 (2019): 2479–2481, 10.1111/all.13869. 31087670

[9] A. Ariano , M. D'Apolito , M. Bova , et al., “A Myoferlin gain‐of‐function Variant Associates With a New Type of Hereditary Angioedema,” Allergy 75, no. 11 (2020): 2989–2992, 10.1111/all.14454. 32542751

[10] K. Bork , K. Wulff , B. S. Mohl , et al., “Novel Hereditary Angioedema Linked With a Heparan Sulfate 3‐O‐sulfotransferase 6 Gene Mutation,” Journal of Allergy and Clinical Immunology 148, no. 4 (2021): 1041–1048, 10.1016/j.jaci.2021.01.011. 33508266

[11] M. D'Apolito , R. Santacroce , D. O. Vazquez , et al., “DAB2IP Associates With Hereditary Angioedema: Insights Into the Role of VEGF Signaling in HAE Pathophysiology,” Journal of Allergy and Clinical Immunology 154, no. 3 (2024): 698–706, 10.1016/j.jaci.2024.05.017. 38823490

[12] D. Vincent , F. Parsopoulou , L. Martin , et al., “Hereditary Angioedema With Normal C1 Inhibitor Associated With Carboxypeptidase N Deficiency,” Journal of Allergy and Clinical Immunology: Global 3, no. 2 (2024): 100223, 10.1016/j.jacig.2024.100223. 38445235 PMC10912455

[13] C. Radojicic and J. Anderson , “Hereditary Angioedema With Normal C1 Esterase Inhibitor: Current Paradigms and Clinical Dilemmas,” Allergy and Asthma Proceedings 45, no. 3 (2024): 147–157, 10.2500/aap.2024.45.240010. 38755781

[14] A. P. Kaplan , D. Pawaskar , and J. Chiao , “C1 Inhibitor Activity and Angioedema Attacks in Patients With Hereditary Angioedema,” Journal of Allergy and Clinical Immunology: In Practice 8, no. 3 (2020): 892–900, 10.1016/j.jaip.2019.10.003. 31655295

[15] H. Longhurst and K. Bork , “Hereditary Angioedema: An Update on Causes, Manifestations and Treatment,” British Journal of Hospital Medicine 80, no. 7 (2019): 391–398, 10.12968/hmed.2019.80.7.391. 31283393

[16] M. Magerl , H. Gothe , S. Krupka , A. Lachmann , and C. Ohlmeier , “A Germany‐Wide Survey Study on the Patient Journey of Patients With Hereditary Angioedema,” Orphanet Journal of Rare Diseases 15, no. 1 (2020): 221, 10.1186/s13023-020-01506-5. 32843072 PMC7448463

[17] K. Bork , J. Hardt , and G. Witzke , “Fatal Laryngeal Attacks and Mortality in Hereditary Angioedema due to C1‐INH Deficiency,” Journal of Allergy and Clinical Immunology 130, no. 3 (2012): 692–697, 10.1016/j.jaci.2012.05.055. 22841766

[18] F. G. Minafra , T. R. Gonçalves , T. M. Alves , and J. A. Pinto , “The Mortality From Hereditary Angioedema Worldwide: A Review of the Real‐World Data Literature,” Clinical Reviews in Allergy and Immunology 62, no. 1 (2022): 232–239, 10.1007/s12016-021-08897-8. 34687444

[19] S. C. Christiansen , D. K. Davis , A. J. Castaldo , and B. L. Zuraw , “Pediatric Hereditary Angioedema: Onset, Diagnostic Delay, and Disease Severity,” Clinical Pediatrics 55, no. 10 (2016): 935–942, 10.1177/0009922815616886. 26581355

[20] A. Zanichelli , M. Magerl , H. Longhurst , V. Fabien , and M. Maurer , “Hereditary Angioedema With C1 Inhibitor Deficiency: Delay in Diagnosis in Europe,” Allergy, Asthma and Clinical Immunology 9, no. 1 (2013): 29, 10.1186/1710-1492-9-29. PMC3751114 23937903

[21] A. K. Jindal , A. Reshef , H. Longhurst , et al., “Mitigating Disparity in Health‐Care Resources Between Countries for Management of Hereditary Angioedema,” Clinical Reviews in Allergy and Immunology 61, no. 1 (2021): 84–97, 10.1007/s12016-021-08854-5. 34003432 PMC8282575

[22] E. Y. Lee , J. Hsieh , T. Caballero , et al., “Demographic and Clinical Characteristics of Patients With Hereditary Angioedema in Canada,” Annals of Allergy, Asthma, & Immunology 128, no. 1 (2022): 89–94.e81, 10.1016/j.anai.2021.07.015. 34298173

[23] M. Maurer , W. Aberer , T. Caballero , et al., “The Icatibant Outcome Survey: 10 Years of Experience With Icatibant for Patients With Hereditary Angioedema,” Clinical and Experimental Allergy 52, no. 9 (2022): 1048–1058, 10.1111/cea.14206. 35861129

[24] K. Iwamoto , B. Yamamoto , I. Ohsawa , et al., “The Diagnosis and Treatment of Hereditary Angioedema Patients in Japan: A Patient Reported Outcome Survey,” Allergology International 70, no. 2 (2021): 235–243, 10.1016/j.alit.2020.09.008. 33168485

[25] I. Guryanova , C. Suffritti , D. Parolin , et al., “Hereditary Angioedema due to C1 Inhibitor Deficiency in Belarus: Epidemiology, Access to Diagnosis and Seven Novel Mutations in SERPING1 Gene,” Clinical and Molecular Allergy 19, no. 1 (2021): 3, 10.1186/s12948-021-00141-0. 33827715 PMC8028818

[26] A. Zanichelli , M. Magerl , H. J. Longhurst , et al., “Improvement in Diagnostic Delays Over Time in Patients With Hereditary Angioedema: Findings From the Icatibant Outcome Survey,” Clinical and Translational Allergy 8, no. 1 (2018): 42, 10.1186/s13601-018-0229-4. 30338053 PMC6182796

[27] A. Agostoni , E. Aygören‐Pürsün , K. E. Binkley , et al., “Hereditary and Acquired Angioedema: Problems and Progress: Proceedings of the Third C1 Esterase Inhibitor Deficiency Workshop and Beyond,” supplement, Journal of Allergy and Clinical Immunology 114, no. S3 (2004): S51–S131, 10.1016/j.jaci.2004.06.047. 15356535 PMC7119155

[28] A. Zanichelli , H. J. Longhurst , M. Maurer , et al., “Misdiagnosis Trends in Patients With Hereditary Angioedema From the real‐world Clinical Setting,” Annals of Allergy, Asthma, & Immunology 117, no. 4 (2016): 394–398, 10.1016/j.anai.2016.08.014. 27742086

[29] J. G. Kemp and T. J. Craig , “Variability of Prodromal Signs and Symptoms Associated With Hereditary Angioedema Attacks: A Literature Review,” Allergy and Asthma Proceedings 30, no. 5 (2009): 493–499, 10.2500/aap.2009.30.3278. 19843403

[30] K. Bork , “Pasteurized and Nanofiltered, plasma‐derived C1 Esterase Inhibitor Concentrate for the Treatment of Hereditary Angioedema,” Immunotherapy 6, no. 5 (2014): 533–551, 10.2217/imt.14.33. 24635050

[31] A. H. Schmaier , “The Contact Activation and kallikrein/kinin Systems: Pathophysiologic and Physiologic Activities,” Journal of Thrombosis and Haemostasis 14, no. 1 (2016): 28–39, 10.1111/jth.13194. 26565070

[32] B. L. Zuraw , “Clinical Practice. Hereditary Angioedema,” New England Journal of Medicine 359, no. 10 (2008): 1027–1036, 10.1056/NEJMcp0803977. 18768946

[33] D. Csuka , N. Veszeli , L. Varga , Z. Prohászka , and H. Farkas , “The Role of the Complement System in Hereditary Angioedema,” Molecular Immunology 89 (2017): 59–68, 10.1016/j.molimm.2017.05.020. 28595743

[34] H. Wang and M. Liu , “Complement C4, Infections, and Autoimmune Diseases,” Frontiers in Immunology 12 (2021): 694928, 10.3389/fimmu.2021.694928. 34335607 PMC8317844

[35] D. M. Cohn and T. Renné , “Targeting Factor Xiia for Therapeutic Interference With Hereditary Angioedema,” Journal of Internal Medicine 296, no. 4 (2024): 311–326, 10.1111/joim.20008. 39331688

[36] R. S. Petersen , L. M. Fijen , M. Levi , and D. M. Cohn , “Hereditary Angioedema: The Clinical Picture of Excessive Contact Activation,” Seminars in Thrombosis and Hemostasis 50, no. 7 (2024): 978–988, 10.1055/s-0042-1758820. 36417927 PMC11407848

[37] D. Levy , T. Craig , P. K. Keith , G. Krishnarajah , R. Beckerman , and S. Prusty , “Co‐Occurrence Between C1 Esterase Inhibitor Deficiency and Autoimmune Disease: A Systematic Literature Review,” Allergy, Asthma and Clinical Immunology 16, no. 1 (2020): 41, 10.1186/s13223-020-00437-x. PMC7254644 32514272

[38] U. O. Wenzel , C. Kemper , and M. Bode , “The Role of Complement in Arterial Hypertension and Hypertensive End Organ Damage,” British Journal of Pharmacology 178, no. 14 (2021): 2849–2862, 10.1111/bph.15171. 32585035 PMC10725187

[39] B. L. Sundler , B. Persson , D. Aronsson , L. Skattum , P. Nordenfelt , and A. Egesten , “Comorbidities in Hereditary Angioedema—A Population‐Based Cohort Study,” Clinical and Translational Allergy 12, no. 3 (2022): e12135, 10.1002/clt2.12135. 35344299 PMC8967273

[40] S. L. Coss , D. Zhou , G. T. Chua , et al., “The Complement System and Human Autoimmune Diseases,” Journal of Autoimmunity 137 (2023): 102979, 10.1016/j.jaut.2022.102979. 36535812 PMC10276174

[41] A. C. Macedo and L. Isaac , “Systemic Lupus Erythematosus and Deficiencies of Early Components of the Complement Classical Pathway,” Frontiers in Immunology 7 (2016): 55, 10.3389/fimmu.2016.00055. 26941740 PMC4764694

[42] E. Willems , L. Lorés‐Motta , A. Zanichelli , et al., “Quantitative Multiplex Profiling of the Complement System to Diagnose Complement‐Mediated Diseases,” Clinical & Translational Immunology 9, no. 12 (2020): e1225, 10.1002/cti2.1225. 33318796 PMC7724921

[43] S. P. Grover , L. Sundler Björkman , A. Egesten , S. Moll , and N. Mackman , “Hereditary Angioedema Is Associated With an Increased Risk of Venous Thromboembolism,” Journal of Thrombosis and Haemostasis 20, no. 11 (2022): 2703–2706, 10.1111/jth.15870. 36053174 PMC12541594

[44] S. G. G. Atay , D. Baykız , İD. Toprak , et al., “C1 Inhibitor Deficient Hereditary Angioedema Is Related to Endothelial Dysfunction in Young Adult and Middle‐Aged Patients,” Clinical and Translational Allergy 15, no. 6 (2025): e70076, 10.1002/clt2.70076. 40551525 PMC12185901

[45] S. C. Christiansen , J. Wilmot , A. J. Castaldo , and B. L. Zuraw , “The US Hereditary Angioedema Association Scientific Registry: Hereditary Angioedema Demographics, Disease Severity, and Comorbidities,” Annals of Allergy, Asthma, & Immunology 131, no. 6 (2023): 766–774e768, 10.1016/j.anai.2023.08.012. 37619776

[46] S. P. Grover , O. Snir , K. Hindberg , et al., “High Plasma Levels of C1‐inhibitor Are Associated With Lower Risk of Future Venous Thromboembolism,” Journal of Thrombosis and Haemostasis 21, no. 7 (2023): 1849–1860, 10.1016/j.jtha.2023.03.024. 37003465 PMC11112258

[47] H. Farkas , D. Levy , D. Supina , M. Berger , S. Prusty , and M. Fridman , “Hereditary Angioedema C1‐Esterase Inhibitor Replacement Therapy and Coexisting Autoimmune Disorders: Findings From a Claims Database,” Allergy, Asthma and Clinical Immunology 16, no. 1 (2020): 42, 10.1186/s13223-020-00439-9. PMC7254637 32514273

[48] M. Magerl , J. Chiao , D. Supina , et al., “Autoimmune Disorders Associated With Hereditary Angioedema: Systematic Literature Review and Findings From USA Claims Database,” Allergy 74 (2019): 272: (Abstract PD0494), 10.1111/all.13959.

[49] F. G. Minafra , L. A. O. Cunha , R. G. S. Mariano , G. A. Goebel , L. S. de Lima , and J. A. Pinto , “Investigation of Mortality of Hereditary Angioedema in a Reference Center in Brazil,” Journal of Allergy and Clinical Immunology: In Practice 10, no. 7 (2022): 1805–1812, 10.1016/j.jaip.2022.04.030. 35526778

[50] D. Brackertz and F. Kueppers , “Hereditary Angioneurotic Oedema,” Lancet 2 (1973): 680, 10.1016/s0140-6736(73)92519-1. 4125654

[51] K. Bork , E. Aygören‐Pürsün , M. Bas , et al., “Guideline: Hereditary Angioedema due to C1 Inhibitor Deficiency,” Allergo Journal International 28, no. 1 (2019): 16–29, 10.1007/s40629-018-0088-5.

[52] K. Bork , “Pasteurized C1 Inhibitor Concentrate in Hereditary Angioedema: Pharmacology, Safety, Efficacy and Future Directions,” Expert Review of Clinical Immunology 4, no. 1 (2008): 13–20, 10.1586/1744666x.4.1.13. 20477582

[53] Sanquin, (2012), https://www.sanquin.org/binaries/content/assets/nl/over‐sanquin/bloedbeeld/bloedbeeld‐nr‐1‐maart‐2012.pdf.

[54] J. J. Hofstra , I. Kleine Budde , E. van Twuyver , et al., “Treatment of Hereditary Angioedema With Nanofiltered C1‐esterase Inhibitor Concentrate (Cetor®): Multi‐center Phase II and III Studies to Assess Pharmacokinetics, Clinical Efficacy and Safety,” Clinical Immunology 142, no. 3 (2012): 280–290, 10.1016/j.clim.2011.11.005. 22197071

[55] J. A. Bernstein , H. H. Li , T. J. Craig , et al., “Indirect Comparison of Intravenous vs. Subcutaneous C1‐inhibitor placebo‐controlled Trials for Routine Prevention of Hereditary Angioedema Attacks,” Allergy, Asthma and Clinical Immunology 15, no. 1 (2019): 13, 10.1186/s13223-019-0328-3. PMC6407188 30899278

[56] A. Zanichelli , G. M. Azin , F. Cristina , R. Vacchini , and T. Caballero , “Safety, Effectiveness, and Impact on Quality of Life of self‐administration With Plasma‐Derived Nanofiltered C1 Inhibitor (Berinert®) in Patients With Hereditary Angioedema: The SABHA Study,” Orphanet Journal of Rare Diseases 13, no. 1 (2018): 51, 10.1186/s13023-018-0797-3. 29631595 PMC5891972

[57] B. L. Zuraw , M. Cicardi , H. J. Longhurst , et al., “Phase II Study Results of a Replacement Therapy for Hereditary Angioedema With Subcutaneous C1‐inhibitor Concentrate,” Allergy 70, no. 10 (2015): 1319–1328, 10.1111/all.12658. 26016741 PMC4755045

[58] CSL Behring LLC , Haegarda. U.S. Food and Drug Administration, (2020): Prescribing Information https://www.fda.gov/media/105611/download.

[59] T. Craig , W. Lumry , M. Cicardi , et al., “Treatment Effect of Switching From Intravenous to Subcutaneous C1‐inhibitor for Prevention of Hereditary Angioedema Attacks: COMPACT Subgroup Findings,” Journal of Allergy and Clinical Immunology: In Practice 7, no. 6 (2019): 2035–2038, 10.1016/j.jaip.2019.01.007. 30660873

[60] T. J. Craig , R. J. Levy , R. L. Wasserman , et al., “Efficacy of Human C1 Esterase Inhibitor Concentrate Compared With Placebo in Acute Hereditary Angioedema Attacks,” Journal of Allergy and Clinical Immunology 124, no. 4 (2009): 801–808, 10.1016/j.jaci.2009.07.017. 19767078

[61] B. L. Zuraw , P. J. Busse , M. White , et al., “Nanofiltered C1 Inhibitor Concentrate for Treatment of Hereditary Angioedema,” New England Journal of Medicine 363, no. 6 (2010): 513–522, 10.1056/NEJMoa0805538. 20818886

[62] H. Longhurst , M. Cicardi , T. Craig , et al., “Prevention of Hereditary Angioedema Attacks With a Subcutaneous C1 Inhibitor,” New England Journal of Medicine 376, no. 12 (2017): 1131–1140, 10.1056/NEJMoa1613627. 28328347

[63] P. E. Hewitt , S. Ijaz , S. R. Brailsford , et al., “Hepatitis E Virus in Blood Components: A Prevalence and Transmission Study in Southeast England,” Lancet 384, no. 9956 (2014): 1766–1773, 10.1016/s0140-6736(14)61034-5. 25078306

[64] T. L. Simon , U. Kalina , R. Laske , S. Mycroft , E. Widmer , and N. J. Roth , “Manufacturing of plasma‐derived C1‐inhibitor Concentrate for Treatment of Patients With Hereditary Angioedema,” Allergy and Asthma Proceedings 41, no. 2 (2020): 99–107, 10.2500/aap.2020.41.190021. 31796151

[65] T. J. Craig , A. K. Bewtra , S. L. Bahna , et al., “C1 Esterase Inhibitor Concentrate in 1085 Hereditary Angioedema Attacks – Final Results of the I.M.P.A.C.T.2 Study,” Allergy 66, no. 12 (2011): 1604–1611, 10.1111/j.1398-9995.2011.02702.x. 21884533

[66] T. Craig , B. Zuraw , H. Longhurst , et al., “Long‐Term Outcomes With Subcutaneous C1‐Inhibitor Replacement Therapy for Prevention of Hereditary Angioedema Attacks,” Journal of Allergy and Clinical Immunology: In Practice 7, no. 6 (2019): 1793.e1792–1802.e1792, 10.1016/j.jaip.2019.01.054. 30772477

[67] K. Bork , J. A. Bernstein , T. Machnig , and T. J. Craig , “Efficacy of Different Medical Therapies for the Treatment of Acute Laryngeal Attacks of Hereditary Angioedema due to C1‐esterase Inhibitor Deficiency,” Journal of Emergency Medicine 50, no. 4 (2016): 567.e561–580.e561, 10.1016/j.jemermed.2015.11.008. 26826769

[68] A. Bygum , K. E. Andersen , and C. S. Mikkelsen , “Self‐Administration of Intravenous C1‐inhibitor Therapy for Hereditary Angioedema and Associated Quality of Life Benefits,” European Journal of Dermatology 19, no. 2 (2009): 147–151, 10.1684/ejd.2008.0603. 19264579

[69] A. Bygum , “Hereditary Angioedema – Consequences of a New Treatment Paradigm in Denmark,” Acta Dermato‐Venereologica 94, no. 4 (2014): 436–441, 10.2340/00015555-1743. 24202369

[70] W. Kreuz , E. Rusicke , I. Martinez‐Saguer , E. Aygören‐Pürsün , C. Heller , and T. Klingebiel , “Home Therapy With Intravenous Human C1‐inhibitor in Children and Adolescents With Hereditary Angioedema,” Transfusion 52, no. 1 (2012): 100–107, 10.1111/j.1537-2995.2011.03240.x. 21756262

[71] K. Bork , J. Hardt , P. Staubach‐Renz , and G. Witzke , “Risk of Laryngeal Edema and Facial Swellings After Tooth Extraction in Patients With Hereditary Angioedema With and Without Prophylaxis With C1 Inhibitor Concentrate: A Retrospective Study,” Oral Surgery, Oral Medicine, Oral Pathology, Oral Radiology & Endodontics 112, no. 1 (2011): 58–64, 10.1016/j.tripleo.2011.02.034. 21601496

[72] E. Aygören‐Pürsün , I. Martinez Saguer , W. Kreuz , T. Klingebiel , and D. Schwabe , “Risk of Angioedema Following Invasive or Surgical Procedures in HAE Type I and II – The Natural History,” Allergy 68, no. 8 (2013): 1034–1039, 10.1111/all.12186. 23968383 PMC4223932

[73] H. Farkas , Z. Zotter , D. Csuka , et al., “Short‐Term Prophylaxis in Hereditary Angioedema due to Deficiency of the C1‐inhibitor – A Long‐Term Survey,” Allergy 67, no. 12 (2012): 1586–1593, 10.1111/all.12032. 23025435

[74] M. Magerl , M. Frank , W. Lumry , et al., “Short‐Term Prophylactic Use of C1‐inhibitor Concentrate in Hereditary Angioedema: Findings From an International Patient Registry,” Annals of Allergy, Asthma, & Immunology 118, no. 1 (2017): 110–112, 10.1016/j.anai.2016.10.006. 27865714

[75] G. Gavigan , W. H. Yang , S. Santucci , R. Harrison , and J. Karsh , “The Prophylactic Use of C1 Inhibitor in Hereditary Angioedema Patients Undergoing Invasive Surgical Procedures: A Retrospective Study,” Allergy, Asthma and Clinical Immunology 10, no. 1 (2014): 17, 10.1186/1710-1492-10-17. PMC4000454 24772176

[76] A. Zanichelli , M. Ghezzi , I. Santicchia , et al., “Short‐Term Prophylaxis in Patients With Angioedema due to C1‐inhibitor Deficiency Undergoing Dental Procedures: An Observational Study,” PLoS One 15, no. 3 (2020): e0230128, 10.1371/journal.pone.0230128. 32163480 PMC7067439

[77] B. L. Zuraw and I. Kalfus , “Safety and Efficacy of Prophylactic Nanofiltered C1‐inhibitor in Hereditary Angioedema,” American Journal of Medicine 125, no. 938 (2012): e931–e937, 10.1016/j.amjmed.2012.02.020. 22800873

[78] I. Martinez‐Saguer , M. Cicardi , C. Suffritti , et al., “Pharmacokinetics of Plasma‐Derived C1‐esterase Inhibitor After Subcutaneous Versus Intravenous Administration in Subjects With Mild or Moderate Hereditary Angioedema: The PASSION Study,” Transfusion 54, no. 6 (2014): 1552–1561, 10.1111/trf.12501. 24266596 PMC4215596

[79] D. Pawaskar , M. A. Tortorici , B. Zuraw , et al., “Population Pharmacokinetics of Subcutaneous C1‐inhibitor for Prevention of Attacks in Patients With Hereditary Angioedema,” Clinical and Experimental Allergy 48, no. 10 (2018): 1325–1332, 10.1111/cea.13220. 29998524

[80] T. Craig , H. Feuersenger , I. Pragst , and J. Dang , “Prophylactic Therapy With Subcutaneous C1‐inhibitor Is Associated With Sustained Symptom Control in Patients With Hereditary Angioedema,” Allergy and Asthma Proceedings 43, no. 3 (2022): 202–208, 10.2500/aap.2022.43.220016. 35524357

[81] M. Pedrosa , T. Lobera , C. Panizo , J. Jurado , and T. Caballero , “Long‐Term Prophylaxis With C1‐inhibitor Concentrate in Patients With Hereditary Angioedema,” Journal of Investigational Allergology & Clinical Immunology 24, no. 4 (2014): 271–273, https://www.jiaci.org/summary/vol24‐issue4‐num1138. 25219111

[82] W. R. Lumry , T. Craig , B. Zuraw , et al., “Health‐Related Quality of Life With Subcutaneous C1‐inhibitor for Prevention of Attacks of Hereditary Angioedema,” Journal of Allergy and Clinical Immunology: In Practice 6, no. 5 (2018): 1733–1741.e1733, 10.1016/j.jaip.2017.12.039. 29391286

[83] W. R. Lumry , B. Zuraw , M. Cicardi , et al., “Long‐Term Health‐Related Quality of Life in Patients Treated With Subcutaneous C1‐inhibitor Replacement Therapy for the Prevention of Hereditary Angioedema Attacks: Findings From the COMPACT open‐label Extension Study,” Orphanet Journal of Rare Diseases 16, no. 1 (2021): 86, 10.1186/s13023-020-01658-4. 33588897 PMC7885603

[84] J. Anderson , D. S. Levy , W. Lumry , P. Koochaki , S. Lanar , and H. Henry Li , “Letting the Patients Speak: An in‐depth, Qualitative Research‐Based Investigation of Factors Relevant to health‐related Quality of Life in real‐world Patients With Hereditary Angioedema Using Subcutaneous C1 Inhibitor Replacement Therapy,” Allergy, Asthma and Clinical Immunology 17, no. 1 (2021): 60, 10.1186/s13223-021-00550-5. PMC8237414 34176500

[85] A. J. Castaldo , C. Jervelund , D. Corcoran , H. B. Boysen , S. C. Christiansen , and B. L. Zuraw , “Assessing the Cost and quality‐of‐life Impact of on‐demand‐only Medications for Adults With Hereditary Angioedema,” Allergy and Asthma Proceedings 42, no. 2 (2021): 108–117, 10.2500/aap.2021.42.200127. 33581742 PMC8133018

[86] M. Maurer and M. Magerl , “Differences and Similarities in the Mechanisms and Clinical Expression of Bradykinin‐Mediated Vs. Mast Cell‐Mediated Angioedema,” Clinical Reviews in Allergy and Immunology 61, no. 1 (2021): 40–49, 10.1007/s12016-021-08841-w. 33534062 PMC8282544

[87] M. Cicardi , W. Aberer , A. Banerji , et al., “Classification, Diagnosis, and Approach to Treatment for Angioedema: Consensus Report From the Hereditary Angioedema International Working Group,” Allergy 69, no. 5 (2014): 602–616, 10.1111/all.12380. 24673465

[88] H. Farkas , K. V. Kőhalmi , N. Veszeli , Z. Zotter , K. Várnai , and L. Varga , “Risk of Thromboembolism in Patients With Hereditary Angioedema Treated With Plasma‐Derived C1‐inhibitor,” Allergy and Asthma Proceedings 37, no. 2 (2016): 164–170, 10.2500/aap.2016.37.3933. 26802388

[89] CSL Behring GmbH , Berinert 500/1500. Summary of Product Characteristics, (2021), https://labeling.cslbehring.com/SMPC/EU/Berinert/EN/Berinert‐500‐1500‐SPC.pdf.

[90] A. Reshef , D. Levy , H. Longhurst , et al., “Effects of Continuous Plasma‐Derived Subcutaneous C1‐Esterase Inhibitor on Coagulation and Fibrinolytic Parameters,” Thrombosis and Haemostasis 121, no. 5 (2021): 690–693, 10.1055/s-0040-1721147. 33202446

[91] P. J. Busse , S. C. Christiansen , M. A. Riedl , et al., “US HAEA Medical Advisory Board 2020 Guidelines for the Management of Hereditary Angioedema,” Journal of Allergy and Clinical Immunology: In Practice 9, no. 1 (2021): 132.e133,–150.e133, 10.1016/j.jaip.2020.08.046. 32898710

[92] CSL Behring LLC , Berinert 500. U.S. Food and Drug Administration, (2021): Prescribing Information, https://www.fda.gov/media/77803/download.

[93] Dyax Corp , A Takeda Company. Kalbitor. U.S. Food and Drug Administration, (2020): Prescribing Information, https://www.accessdata.fda.gov/drugsatfda_docs/label/2020/125277s081lbl.pdf.

[94] Pharming Group N.V. Ruconest , U.S. Food and Drug Administration, (2014): Prescribing Information, https://www.fda.gov/media/89212/download.

[95] Pharming Group N.V ., Ruconest. European Medicines Agency. Summary of Product Characteristics, (2021), https://www.ema.europa.eu/en/documents/product‐information/ruconest‐epar‐product‐information_en.pdf.

[96] Takeda Manufacturing Austria AG , Cinryze. European Medicines Agency, Summary of Product Characteristics, (2016), https://www.ema.europa.eu/en/documents/product‐information/cinryze‐epar‐product‐information_en.pdf.

[97] Takeda Pharmaceuticals America Inc , Firazyr. U.S. Food and Drug Administration, (2021): Prescribing Information, https://www.accessdata.fda.gov/drugsatfda_docs/label/2022/022150Orig1s014lbl.pdf.

[98] Takeda Pharmaceuticals International AG Ireland Branch , Firazyr. European Medicines Agency, Summary of Product Characteristics, (2013), https://www.ema.europa.eu/en/documents/product‐information/firazyr‐epar‐product‐information_en.pdf.

[99] BioCryst Ireland Limited. Orladeyo. European Medicines Agency , Summary of Product Characteristics, (2021), https://www.ema.europa.eu/en/documents/product‐information/orladeyo‐epar‐product‐information_en.pdf.

[100] BioCryst Pharmaceuticals Inc , Orladeyo. U.S. Food and Drug Administration, (2023): Prescribing Information, https://www.accessdata.fda.gov/drugsatfda_docs/label/2023/214094s002lbl.pdf.

[101] CSL Behring GmbH , Berinert 2000/3000. Summary of Product Characteristics, (2021), https://labeling.cslbehring.com/SMPC/EU/Berinert/EN/Berinert‐2000‐3000‐SPC.pdf.

[102] Dyax Corp , Takhzyro. U.S. Food and Drug Administration, (2018): Prescribing Information, https://www.accessdata.fda.gov/drugsatfda_docs/label/2018/761090s001lbl.pdf.

[103] Takeda Pharmaceuticals International AG Ireland Branch , Takhzyro. European Medicines Agency, Summary of Product Characteristics, (2023), https://www.ema.europa.eu/en/documents/product‐information/takhzyro‐epar‐product‐information_en.pdf.

[104] ViroPharma Biologics LLC , Cinryze. U.S. Food and Drug Administration, Prescribing Information, (2022), https://www.fda.gov/media/75907/download.

[105] T. J. Craig , A. Reshef , H. H. Li , et al., “Efficacy and Safety of Garadacimab, a Factor Xiia Inhibitor for Hereditary Angioedema Prevention (VANGUARD): A Global, Multicentre, Randomised, double‐blind, placebo‐controlled, Phase 3 Trial,” Lancet 401, no. 10382 (2023): 1079–1090, 10.1016/s0140-6736(23)00350-1. 36868261

[106] CSL Behring LLC , ANDEMBRY. U.S. Food and Drug Administration, (2025): Prescribing Information, https://www.accessdata.fda.gov/drugsatfda_docs/label/2025/761367s000lbl.pdf.

[107] M. A. Riedl , R. Tachdjian , W. R. Lumry , et al., “Efficacy and Safety of Donidalorsen for Hereditary Angioedema,” New England Journal of Medicine 391, no. 1 (2024): 21–31, 10.1056/NEJMoa2402478. 38819395

[108] Ionis Pharmaceuticals Inc , DAWNZERA. U.S. Food and Drug Administration, (2025): Prescribing Information, https://ionis.com/medicines/DAWNZERA/DAWNZERA‐FPI.pdf.

[109] M. A. Riedl , H. Farkas , E. Aygören‐Pürsün , et al., “Oral Sebetralstat for on‐demand Treatment of Hereditary Angioedema Attacks,” New England Journal of Medicine 391, no. 1 (2024): 32–43, 10.1056/NEJMoa2314192. 38819658

[110] KalVista Pharmaceuticals Inc , EKTERLY. U.S. Food and Drug Administration, (2025): Prescribing Information, https://www.accessdata.fda.gov/drugsatfda_docs/label/2025/219301s000lbl.pdf.

[111] M. Maurer , E. Aygören‐Pürsün , A. Banerji , et al., “Consensus on Treatment Goals in Hereditary Angioedema: A Global Delphi Initiative,” Journal of Allergy and Clinical Immunology 148, no. 6 (2021): 1526–1532, 10.1016/j.jaci.2021.05.016. 34048855

[112] K. Bork , K. Wulff , G. Witzke , and J. Hardt , “Treatment for Hereditary Angioedema With Normal C1‐INH and Specific Mutations in the F12 Gene (HAE‐FXII),” Allergy 72, no. 2 (2017): 320–324, 10.1111/all.13076. 27905115

[113] B. Geng and M. A. Riedl , “HAE Update: Special Considerations in the Female Patient With Hereditary Angioedema,” Allergy and Asthma Proceedings 34, no. 1 (2013): 13–18, 10.2500/aap.2013.34.3635. 23406930

[114] K. Bork , K. Wulff , G. Witzke , T. Machnig , and J. Hardt , “Treatment of Patients With Hereditary Angioedema With the c.988A> G (p.Lys330Glu) Variant in the Plasminogen Gene,” Orphanet Journal of Rare Diseases 15, no. 1 (2020): 52, 10.1186/s13023-020-1334-8. 32066472 PMC7026952

[115] P. Triggianese , R. Senter , A. Petraroli , et al., “Pregnancy in Women With Hereditary Angioedema due to C1‐inhibitor Deficiency: Results From the ITACA Cohort Study on Outcome of Mothers and Children With in Utero Exposure to plasma‐derived C1‐inhibitor,” Frontiers of Medicine 9 (2022): 930403, 10.3389/fmed.2022.930403. PMC9515414 36186797

[116] I. Martinez‐Saguer , E. Rusicke , E. Aygören‐Pürsün , et al., “Characterization of Acute Hereditary Angioedema Attacks During Pregnancy and Breast‐Feeding and Their Treatment With C1 Inhibitor Concentrate,” American Journal of Obstetrics and Gynecology 203 (2010): 131.e131–137.e131, 10.1016/j.ajog.2010.03.003. 20471627

[117] W. Lumry , M. E. Manning , D. S. Hurewitz , et al., “Nanofiltered C1‐esterase Inhibitor for the Acute Management and Prevention of Hereditary Angioedema Attacks due to C1‐inhibitor Deficiency in Children,” Jornal de Pediatria 162, no. 5 (2013): 1017–1022: e1011–1012, 10.1016/j.jpeds.2012.11.030. 23312695

[118] M. Cicardi , K. Bork , T. Caballero , et al., “Evidence‐Based Recommendations for the Therapeutic Management of Angioedema Owing to Hereditary C1 Inhibitor Deficiency: Consensus Report of an International Working Group,” Allergy 67, no. 2 (2012): 147–157, 10.1111/j.1398-9995.2011.02751.x. 22126399

[119] J. Fox , A. B. Vegh , I. Martinez‐Saguer , et al., “Safety of a C1‐inhibitor Concentrate in Pregnant Women With Hereditary Angioedema,” Allergy and Asthma Proceedings 38, no. 3 (2017): 216–221, 10.2500/aap.2017.38.4038. 28441992

[120] I. Chair , G. Lacuesta , C. M. Nash , and V. Cook , “A Challenging Diagnosis: Hereditary Angioedema Presenting During Pregnancy,” Canadian Medical Association Journal 194, no. 37 (2022): E1283–e1287, 10.1503/cmaj.220604. 36162836 PMC9512162

[121] D. S. Levy , H. Farkas , M. A. Riedl , et al., “Long‐Term Efficacy and Safety of Subcutaneous C1‐inhibitor in Women With Hereditary Angioedema: Subgroup Analysis From an Open‐label Extension of a Phase 3 Trial,” Allergy, Asthma and Clinical Immunology 16, no. 1 (2020): 8, 10.1186/s13223-020-0409-3. PMC7001333 32042283

[122] H. Farkas , K. V. Kőhalmi , B. Visy , N. Veszeli , and L. Varga , “Clinical Characteristics and Safety of plasma‐derived C1‐inhibitor Therapy in Children and Adolescents With Hereditary Angioedema—A long‐term Survey,” Journal of Allergy and Clinical Immunology: In Practice 8, no. 7 (2020): 2379–2383, 10.1016/j.jaip.2020.02.043. 32198128

[123] A. Bygum , I. Martinez‐Saguer , M. Bas , et al., “Use of a C1 Inhibitor Concentrate in Adults ≥ 65 Years of Age With Hereditary Angioedema: Findings From the International Berinert® (C1‐INH) Registry,” Drugs Aging 33, no. 11 (2016): 819–827, 10.1007/s40266-016-0403-0. 27699634 PMC5107191

[124] J. A. Bernstein , L. Schwartz , W. Yang , et al., “Long‐Term Safety and Efficacy of Subcutaneous C1‐inhibitor in Older Patients With Hereditary Angioedema,” Annals of Allergy, Asthma, & Immunology 125, no. 3 (2020): 334–340.e331, 10.1016/j.anai.2020.05.015. 32445670

[125] I. Martinez‐Saguer , K. Bork , T. Latysheva , et al., “Plasma‐Derived C1 Esterase Inhibitor Pharmacokinetics and Safety in Patients With Hereditary Angioedema,” Journal of Allergy and Clinical Immunology: Global 3, no. 1 (2024): 100178, 10.1016/j.jacig.2023.100178. 38033485 PMC10684372

[126] Octapharma , Study of IV Human plasma‐derived C1 Esterase Inhibitor Concentrate in Patients With Congenital C1‐INH Deficiency for Treatment and Pre‐procedure Preventing of Acute Hereditary Angioedema Attacks, (2024), https://clinicaltrials.gov/study/NCT06361537.

